# Supplementary material for: The power of past performance in multidimensional supplier evaluation and supplier selection: Debiasing anchoring bias and its knock-on effects
Source: PLoS One. 2024 May 16;19(5):e0303700. doi: 10.1371/journal.pone.0303700 (PMC11098414; doi:10.1371/journal.pone.0303700)
Supplement: Attachment — Submitted filename: Response to Reviewers.docx [file pone.0303700.s003.docx]

**Reviewer #1: This research examines how anchoring bias affects managers' assessments of supplier performance and supplier selection and examines the effectiveness of two debiasing techniques.**

Regarding the methodology used, the author carries out a consistency analysis of the proposed questionnaires. In study 1, independent sample t-test is applied to verify the relationship between anchoring and supplier evaluation. Study 2 was a between-subjects online factorial experiment, and applies ANOVA to analyze the effects of two debiasing techniques: consider-the-opposite and mental remapping.
The work is well structured and well-founded, however, I make some suggestions for improving the article.

**My response:** Thank you sincerely for dedicating your time and efforts to reviewing our manuscript. We greatly appreciate your insightful comments, all of which have been carefully addressed in the revised version of the manuscript.

1. The summary is good, but its structure could be improved, as the author presents the objective of the research, addresses the methodology, presents some results and talks again about the research topic. I suggest that the author follows a structured order to present the study summary, presenting the research topic, the question that the work intends to answer, the methodology used and finally the results obtained. Furthermore, the summary only mentions Study 1 and Study 2, but does not report the methodology used in these studies. I suggest that the methodology is better reported in the summary, avoiding using acronyms (N).

**My response:** Thank you very much for your time and invaluable feedback, which I truly appreciate. I have taken your suggestions into consideration and revised the summary/abstract accordingly. Additionally, I have included the methodology used in the abstract as per your recommendation.

2. Regarding the results, although the author cites five tables, they are not included in the manuscript, making their analysis impossible.

**My response:** Thank you for your message. I understand that the tables were included in the main text in this revision, specifically in the Result section of Study 2. I appreciate your effort to ensure accessibility by also attaching the tables in Appendix 1 at the end of the letter. Thank you again.

3. The methods were detailed, however it was not explained what types of information about the suppliers' current performance the participants had to carry out the evaluation. This information appears only in the appendices. Given the importance of this data for the replication of the study, I suggest that it be better detailed in the text.

**My response:** Thank you for your valuable comment. I wholeheartedly agree that replication of the study is crucial. In the revised version, I have addressed this concern by providing detailed illustrations of the evaluation process (please see lines 391-416) and the information pertaining to the suppliers' current performance in Figures 1-2 (please refer to lines 405-407 and fig1.tiff and fig2.tiff files). Furthermore, I have revised the methodology section to clarify the types of information about the supplier’s current performance provided to the participants for conducting the evaluation.

Moreover, I would like to highlight that the effects of past performance in one dimension on the current evaluations of suppliers’ performance in multiple dimensions have been successfully replicated in Study 2. As stated in the Results section of Study 2, “*Participants in the high-anchor condition rate the current performance in terms of cost more highly than those in the low-anchor condition (M_high_ _anchor_ = 6.21 vs. M_low_ _anchor_ = 4.58; t(134) = 6.12, p < 0.0005, Cohen’s d = 1.07).*” .

4. I suggest further discussion on whether a high anchor supplier's high current performance score is related to bias rather than current performance. What about the low current performance score of a supplier with a low anchor being related to bias and not current performance. Make it clearer to the reader how the questions in the questionnaires guarantee this relationship.

My response: Thank you once again for your valuable comment. I have duly noted your suggestion, and corresponding changes have been implemented on lines 347-355 and 424-422. If you have any further feedback or concerns, please do not hesitate to let me know. Your input is greatly appreciated.

5. The statistical analysis is adequate. However, I suggest that the author better describes the conduct of statistical analyses, such as normality and t-test assumptions, to provide the reader with a better understanding of the characteristics of the samples. Furthermore, I suggest that the statistical software, version and packages used for statistical analyzes are described.

**My response:** Thanks a lot. This is a great idea. I used SPSS version 29 (which has now been included in the manuscript). I completely followed your advice. And, the revised manuscript discussed and tested if the normality assumption was violated, by showing that both skewness coefficient is between ‐2 to +2 and kurtosis coefficient is between ‐7 to +7. These are consistent with the normality assumption suggested by Bryne (2010) [72] and Hair et al. (2010) [73]. The approach I have taken to test the normality assumption by examining the skewness and kurtosis coefficients is sound and aligns with the guidelines suggested by Bryne (2010) [72] and Hair et al. (2010) [73]. It is important to ensure that your data meet the normality assumption of independent samples t-test.

I also re-examine the data using a conservative approach of the Mann Whitney tests. the findings are consistent with the independent-sample t-tests and planned contrasts used in Studies 1 & 2. The Mann Whitney test results are included in Appendix 2 at the bottom of this response letter **for your reference** (but the Mann Whitney test results were not reported in the manuscript, because the skewness and kurtosis coefficients have shown that the data do not violate the independent sample t-test assumption).

6. The data presented by the author support the conclusions. The questionnaires used are presented, which allow the study to be replicated in other contexts. However, I suggest that access to the questionnaire responses be made available so that the present study can be replicated.

**My response:** Thank you very much. I regret to inform you that the datasets were not locatable. It is imperative that findings from experimental studies are replicable, a sentiment with which I wholeheartedly agree. The datasets have now been made accessible via the PLOS One submission portal, enabling other scholars to access the questionnaire responses.

7. All references are cited in the text and all citations are listed under references. However, it is necessary to check whether the citations are in accordance with the submission guidelines, specifically in lines 233, 234, 250, 422 and 426.

**My response:** Thank you for bringing this to my attention, and I apologise for my oversight. In this revised version, all citations throughout the manuscript adhere to the PLOS One submission guidelines. Please refer to lines 47 and 51 as examples.
**Reviewer #2: When I saw the paper's title, I was looking forward to reading “The power of past performance in multidimensional supplier evaluation and supplier selection: Debiasing anchoring bias and its knock-on effects” by Ricky Siu Wong, PhD.**

The experiment is basically successful and offers interesting results. However, I (currently still) have major reservations about whether these results offer any relevant added value for managers in the context of supplier evaluation.

**My response:** Thank you for dedicating your valuable time to provide such insightful feedback. This research is driven by both a theoretical gap, namely the anchoring effect in multi-dimensional evaluation and de-biasing methods, and a tangible issue faced by a prominent organisation. Upon publication, the organisation with whom we collaborated has agreed to implement the de-biasing methods developed in this research and assess their real-world impacts on their supplier evaluation processes. This collaboration holds the potential to yield a significant and impactful case study in the future.

It is noteworthy that both de-biasing techniques proposed in this research offer cost-effective decision aids for mitigating the effects of low and high anchors on supplier evaluation and selection processes. Additionally, an essential practical implication lies in the ripple effects of anchoring on other attributes under evaluation. In the revised manuscript, particular emphasis has been placed on highlighting these practical implications in both the Introduction and Discussion sections (see lines 70-75, 86-95, 107-112, 651-654, 663-666, 677-678, 695-700, 710-723).

Another significant practical contribution concerns the adaptation and modification of Bahník et al.'s (2019) debiasing method for use in multi-dimensional supplier evaluation. This study introduces a novel approach utilising the extreme points of the Likert scale, a method not previously tested in the literature. This innovative approach offers substantial added value for managers engaged in supplier evaluation or similar evaluative tasks across various domains. The Discussion section places particular emphasis on these practical implications (see lines 710-723).

*R2’s comment:* The topic of the article is highly interdisciplinary. Relevant areas include psychology, judgment and decision-making, and supplier evaluation. In my opinion, all sub-areas should be adequately considered. However, my impression is that in this article, this is only the case to a very limited extent in the third area - supplier evaluation:

**My response**: Thanks a lot for your feedback. Please note that psychology and judgment and decision-making (JDM) were indeed discussed in the initial paragraphs under "Theoretical Background and Hypotheses." Recent JDM literature, such as the work by Bahník et al. (2019) [21], was utilised to elucidate the underlying mechanisms through which anchoring influences judgments within a supplier evaluation context (please refer to lines 183-212). Furthermore, both this section and the Discussion have been revised to address your concern (see lines 687-694 for specific examples).

I greatly appreciate your invaluable feedback, which undoubtedly contributes to the strengthening of the paper. The literature section has been enhanced by incorporating JDM and psychology studies (please refer to lines 73-75, 136-211). Additionally, in the revised Discussion section, the relevance of our findings to the fields of JDM and psychology is expounded upon. For instance, the asymmetrical difficulties encountered in de-biasing high versus low anchors offer avenues for future research across all relevant disciplines. Future studies should consider employing a combination of de-biasing methods. The empirical findings from this research provide further support for the scale of distortion theoretical framework regarding the observed anchoring effect. Importantly, the findings suggest that low and high anchors may operate somewhat differently. To the best of my knowledge, this study represents the first investigation into whether these methods could mitigate anchoring's knock-on effects in multi-dimensional evaluation scenarios. These findings also pave the way for future research within the realms of JDM and psychology.

1.) The article does not take up the latest work in the field of behavioral OR,

**My response:** Thanks a lot for this important comment. I take your advice seriously and have thoroughly revised the entire manuscript to demonstrate a mastery of the literature. In doing so, the following behavioural studies have been discussed and cited:

7.	Bendoly E, Donohue K, Schultz KL. Behavior in operations management: Assessing recent findings and revisiting old assumptions. Journal of operations management. 2006;24(6):737-52.

10.	Perera HN, Fahimnia B, Tokar T. Inventory and ordering decisions: a systematic review on research driven through behavioral experiments. International Journal of Operations & Production Management. 2020;40(7/8):997-1039.

11.	Bendoly E, Croson R, Goncalves P, Schultz K. Bodies of knowledge for research in behavioral operations. Production and operations management. 2010;19(4):434-52.

12.	Croson R, Schultz K, Siemsen E, Yeo M. Behavioral operations: the state of the field. Journal of Operations Management. 2013;31(1-2):1-5.

13.	Kull TJ, Oke A, Dooley KJ. Supplier selection behavior under uncertainty: contextual and cognitive effects on risk perception and choice. Decision Sciences. 2014;45(3):467-505.

14.	Schiffels S, Fliedner T, Kolisch R. Human behavior in project portfolio selection: Insights from an experimental study. Decision Sciences. 2018;49(6):1061-87.

15.	Tokar T, Aloysius JA, Waller MA. Supply chain inventory replenishment: The debiasing effect of declarative knowledge. Decision Sciences. 2012;43(3):525-46.

16.	Yamini S. Behavioural operations management: trends and insights. Emerging Frontiers in Operations and Supply Chain Management: Theory and Applications. 2021:233-49.

17.	Zhan Y, Chung L, Lim MK, Ye F, Kumar A, Tan KH. The impact of sustainability on supplier selection: A behavioural study. International Journal of Production Economics. 2021;236:108-18.

19.	Moritz BB, Hill AV, Donohue KL. Individual differences in the newsvendor problem: Behavior and cognitive reflection. Journal of Operations Management. 2013;31(1-2):72-85.

20.	Wong RS. An experimental investigation of attribute-framing effects on ordering decisions in dual sourcing: Role of attention to suppliers’ information. International Journal of Operations and Production Management. 2023.

25.	D'Urso D, Di Mauro C, Chiacchio F, Compagno L. A behavioural analysis of the newsvendor game: Anchoring and adjustment with and without demand information. Computers & Industrial Engineering. 2017;111:552-62.

29.	Davis AM, Hyndman K. Multidimensional bargaining and inventory risk in supply chains: An experimental study. Management Science. 2019;65(3):1286-304.

31.	Doyle J, Ojiako U, Marshall A, Dawson I, Brito M. The anchoring heuristic and overconfidence bias among frontline employees in supply chain organizations. Production Planning & Control. 2021;32(7):549-66.

74.	Schultz KL, Robinson LW, Thomas LJ, Schultz J, McClain JO. The use of framing in inventory decisions. Production and Operations Management. 2018;27(1):49-57.

75.	Villa S, Castañeda JA. A behavioural investigation of power and gender heterogeneity in operations management under uncertainty. Management Research Review. 2020;43(6):753-71.

2.) the motivation (in your Realism Check) is based on anecdotal relevance and was not derived from the literature.

My response: I am sorry that I did not make it sufficiently clear in the original manuscript, and the motivation is also supported by the relevant literature and the real problems faced by a large organisation. The literature that supports my realism check has now been included in the relevant section (please see below for the citations).

20.	Wong RS. An experimental investigation of attribute-framing effects on ordering decisions in dual sourcing: Role of attention to suppliers’ information. International Journal of Operations and Production Management. 2023.

65.	Rungtusanatham M, Wallin C, Eckerd S. The vignette in a scenario‐based role‐playing experiment. Journal of Supply Chain Management. 2011;47(3):9-16.

66.	Golgeci I, Ali I, Bozkurt S, Gligor DM, Arslan A. The impact of corporate support programs on environmental and social innovation: empirical insights from the food and beverage industry. International Journal of Operations & Production Management. 2022 (ahead-of-print).

67.	Bachrach DG, Bendoly E. Rigor in behavioral experiments: A basic primer for supply chain management researchers. Journal of Supply Chain Management. 2011;47(3):5-8.

3.) the evaluation problem, central to the studies, appears to be relatively remote from practice.

**My response:** Thank you very much for your specific comment. As recommended by the literature [1,2,3,79], multi-dimensional evaluation is prevalent in supply chain management (SCM). To ensure that the evaluation problem remains relevant to real-world practice, a realism check was conducted in collaboration with SCM practitioners. Our research employed the third version of the evaluation task, following consultations with these practitioners. They confirmed that the task in its third iteration encapsulates the essential elements of a supplier evaluation task, such as providing a range of prices and determining the order of information presentation. I have revised the 'Realism Check' section accordingly, incorporating references to the literature you mentioned, as I believe this addition would be beneficial to the readers.

*R2’s comment:* I believe the author is able to solve these issues. Therefore, I recommend a major revision of the paper.

**My response**: Thanks a lot for your encouragement.

In the following, you will find some ideas/questions/thoughts. Please refer to your text if I comment on something you have addressed somewhere in the script (and I did not find it).

1.) Could you please provide a graphical illustration of the studies, including all relevant information, to understand them?

**My response:** Thanks a lot for your amazing input. This certainly helps my article in a much better shape. Yes, this was initially placed in the Appendix. Yes, I agree with your wise suggestion and I have relocated the graphical illustrations of the task from the Appendix to the main text. I concur with your decision, and it's noted that Figures 1 and 2 now appear in the Method section of the revised manuscript (please refer to lines 405 and 407). The figures have been saved in two files: Fig1.tiff and Fig2.tiff.

2.) What was the time interval between the individual activities? Was there a distractor task? If not, why not?

**My response:** Upon reviewing existing anchoring studies, it is apparent that the experimental paradigm did not incorporate a distractor, a characteristic shared by this research as well (please refer to Adomavicius et al., 2013; Bahník et al., 2019; Jung et al., 2016; Thornsteinson et al., 2008; Wong, 2023, for examples). Additionally, following discussions with both the supply chain management (SCM) team and senior management of the company under study, which specialises in the production of Disney and Japanese comic figures (e.g., Dragon Ball), it was collectively decided that the inclusion of a distractor task would significantly compromise the external validity of the study. This decision stemmed from the understanding that staff members typically do not engage in distraction tasks during supplier evaluation processes. As such, the SCM team and senior management deemed it unrealistic to introduce a distractor in this context.

3.) Please explain why you chose 104 or 408 participants.

**My response:** I utilised the *G*Power* software package for sample size estimation, a process now outlined in the main text (please refer to pages 1-222). *G*Power* was employed to determine sample sizes for both Study 1 and Study 2. To complete the sample size estimation, I referenced effect sizes from prior anchoring studies within a similar context, aiming for medium power.

For Study 1, the estimated number of participants required was 50 in each condition, totalling a hundred participants. To account for potential speeders and participants who might fail the attention filter tests, 110 participants were recruited. Following exclusion criteria due to failure to pass the attention test or being identified as speeders, 104 participants remained for analysis in Study 1.

A similar procedure was applied to estimate the required sample size for Study 2, resulting in an estimation of 398 participants. Consequently, 420 participants were recruited, with 406 included in the subsequent analyses. It is important to note that 3 participants failed the attention test, and 11 were identified as speeders.

Furthermore, relevant citations have been incorporated into the text. Please see below for details:

69. Beck, T.W., 2013. The importance of a priori sample size estimation in strength and conditioning research. *The Journal of Strength & Conditioning Research*, 27(8), pp. 2323-2337.

4.) You write in the description for Study 1, “Prior to the task, there was an attention filter question ensuring that participants paid attention to the information provided. They were asked to indicate ‘strongly agree’ in the attention filter question.” Please provide information on how many people were screened out. What about Study 2? I cannot find information about an attention check.

**My response:** Thank you for bringing this to my attention. I regret any confusion caused by the lack of clarity in the manuscript regarding the use of attention filters. The relevant section (Method) has been duly revised to address this oversight. In both studies, attention filters were indeed employed. When a participant failed to pass the attention check, they were unable to progress to the subsequent questions, resulting in a completion rate of less than 100%. Subsequently, such cases were excluded and removed from the analyses. In Study 1, five participants who spent less than one-third of the median completion time were excluded from the study (as speeders). Additionally, one participant who failed to pass the attention test was removed from the analysis (please see lines 371-386). In Study 2, eleven participants were identified as speeders, and three participants failed to pass the attention check, utilising the same attention test and methods of identifying speeders as in Study 1 (please see lines 495-497).

*R2’s comment:* For further clarification, please refer to the Method section, specifically the Participants subsections, in both Study 1 and Study 2.5.) You write concerning the completion time “The mean completion time was 7.24 minutes (S.D. = 9.16). How much time did the faster 50% spend on average”. The SD seems high. An explanation might be that several participants have interrupted their assignment. Could you please provide in your answer to the reviewers a distribution of the completion time? (and if necessary, please comment on speeders and how you have dealt with them)

**My response:** Thank you for your raising this issue. The unusually high standard deviation of completion time can be attributed to a single outlier – one participant who took 93.58 minutes to complete the experiment. Upon investigation, it was discovered that this participant had encountered technical difficulties, specifically internet connectivity issues, which resulted in prolonged time spent on the first page (i.e., informed consent form).

Should this outlier be excluded from the analysis, the mean completion time and standard deviation would become 6.40 minutes and 3.31 minutes, respectively. Importantly, excluding this participant does not alter the findings of our study. Therefore, this participant was retained in the analyses. Regarding your question “how much time did the faster 50% spend on average?”, their mean and median completion time are 4.31 mins and 4.24 mins, respectively. Following suggestions from existing literature, specifically Belliveau and Yakovenko (2022) and Greszki (2014), participants who spent less than one-third of the median completion time were excluded from our study. This approach is consistent with best practices in the field and ensures the integrity of our data analysis. Please kindly refer to the citations below:

*Citations:*

70. Belliveau, J. and Yakovenko, I., 2022. Evaluating and improving the quality of survey data from panel and crowd-sourced samples: A practical guide for psychological research. Experimental and Clinical Psychopharmacology, 30(4), p.400–408.

71. Greszki, R., Meyer, M., & Schoen, H. (2014). The impact of speeding on data quality in nonprobability and freshly recruited probability-based online panels. In M. Callegaro, R. Baker, J. Bethlehem, A. S. Goritz, J. A. Krosnick, & P. J. Lavrakas (Eds.), Online panel research: Data quality perspective (pp. 238–262). Wiley. [https://doi.org/10.1002/978 1118763520.ch11](https://doi.org/10.1002/978%201118763520.ch11)

**Please note that the completion time was measured in seconds in the SPSS file (excluding the participant who spent 93.58 minutes (= 5615 seconds) to complete the experiment):**

6.) I think it is fine. However, please explain why you chose a 9-point scale (and why you chose only to name the extremes).

**My response:** Thank you once again. I made the decision to utilise a 9-point scale without labelling all the ordinal categories based on recommendations from supply chain management (SCM) practitioners who reviewed the experimental tasks. They advised that labelling only the lowest and highest scores would suffice, and indicated that a 9-point scale was commonly employed in similar contexts, aligning with the findings from our realism check in organisations.

7.) In the design of your supplier evaluation, many things must be considered (and commented).

**You do not have to address all of them in this paper. Some of my comments might also be useful for further research:**

a. You write, “Study 1 presented a realistic multidimensional supplier evaluation task.” I have some doubts. Why should cost be evaluated on a Likert Scale in practice? Costs can be measured in USD, EUR, etc.

**My response:** Thank you very much, and I completely agree. Initially, in the original version of the experimental protocol, I held the same belief. However, during the revision of the experimental materials, I was informed that typically, the team responsible for supplier evaluations would be provided with a range of market costs and would assess suppliers using a Likert Scale. The rationale behind this approach is to facilitate the computation of a weighted average score for each supplier, ultimately aiding decision-making.

This aspect is now addressed in the Limitations section, in conjunction with your comment highlighted later in this correspondence regarding the potential bandwidth effect.

b. Why did you put your anchors on the dimension “costs”? Costs are often seen as the most crucial dimension.

**My response:** Thank you for raising this point. I initially held a similar perspective to yours before engaging with the supply chain management (SCM) practitioners. However, it became evident through discussions with them that, in many cases, quality is perceived as more important than cost. The SCM practitioners explained that their operational strategy prioritises differentiation, and they highlighted the significant impact that defective parts can have on operations, often resulting in substantial rework and scrap costs. Conversely, they noted that the cost differences among suppliers are typically not as significant.

This insight, gleaned from the interviews, provides valuable context for my current research and will undoubtedly inform my future studies. I greatly appreciate your comment and the opportunity to incorporate this perspective into my work.

c. Why did you put your anchors on the dimension in the first row? This might bias the participants.

**My response:** Thank you once more for your input. I acknowledge your concern regarding the potential bias introduced by placing the anchors as the first dimension in the evaluation task. However, it's important to note that any such bias would be mitigated by the random assignment of participants to experimental conditions in both studies.

Furthermore, it's crucial to emphasize that our primary interest lies in examining the absolute differences between or across experimental conditions. This consideration has been duly addressed in the limitations section of the manuscript (please refer to lines 740-745), where we discuss the potential implications of this experimental design choice.

d. Your supplier is allocated in Tokyo. This information might bias your participants. Why did you consider this information?

**My response:** Thank you for your thoughtful consideration of this issue. I agree with your assessment that the location of the supplier, while potentially introducing a confounding factor, is unlikely to interfere with the overall findings. This is because our analyses primarily focus on comparing the absolute differences across different experimental conditions, and the random assignment of participants to these conditions helps to mitigate any potential bias.

Moreover, specifying the location of the supplier, particularly in Tokyo where the company manufactures Japanese comic toy figures, serves to enhance the external validity of the research. It provides a realistic context for the evaluation task, thereby bolstering the relevance of our findings to real-world scenarios.

e. You provide in a note the bandwidth of the costs. Why did you do that? Why did you not consider this information for the other dimensions? (In decision-making making, the bandwidth effect has crucial implications)

**My response:** Thanks again. This is now discussed in the Limitation section (please see lines 733-739). The potential bandwidth effect (*although this is how things were conducted in the company*) may have an impact on evaluations. Again, random assignment of participants to conditions and only comparing the absolute differences between conditions should eliminate the bandwidth effect (if any). Your input is invaluable, and it underscores the importance of considering these factors in future studies. Additionally, I agree that this feedback could be beneficial for the organisation to consider in their own practices.

f. How did you choose the values for the other dimensions?

**My response:** Thank you once more for your feedback. To enhance the external validity of my research, I based the values for other dimensions on prior supplier evaluations conducted by the company that was interviewed. This approach ensures that the evaluation task closely reflects real-world scenarios, thereby strengthening the generalisability of our findings.

*R2’s comment:* I hope the authors take up the challenge. Good luck.

**My response:** Thank you very much for your kind words! Finally, I want to sincerely express my gratitude for your time and very insightful comments. Without your hard work and effort, it would not be possible to put the article in a much better shape. Thank you so so much.

**Appendix 1 – Study 1 and Study 2 Statistical Analyses Results**

**Table 1. Effects of anchoring and** **debiasing techniques on evaluation of the cost dimension.**

| Source | *SS* | *df* | *MS* | *F* | *p* |
| --- | --- | --- | --- | --- | --- |
| Anchor | 166.01 | 1 | 166.01 | 85.59 | <0.0005 |
| Debiasing | 10.28 | 2 | 5.14 | 2.65 | 0.072 |
| Interaction | 7.08 | 2 | 3.54 | 1.82 | 0.16 |
| Explained | 184.00 | 5 | 36.80 | 18.97 | <0.0005 |
| Residual | 775.83 | 400 | 1.94 |  |  |
| Total | 12957.00 | 406 |  |  |  |

The anchor variable is binary, taking on values of either 0 or 1, with 0 indicating the low-anchor condition. The debiasing variable is categorical, taking on values of 0, 1 or 2. 0 indicates no debiasing technique; 1 represents the use of consider-the-opposite technique and 2 indicates the use of mental-remapping technique. This categorical distinctions remain consistent across Tables 2 to 5.

**Table 2. Effects of anchoring and debiasing techniques on evaluation of the quality dimension.**

| Source | *SS* | *df* | *MS* | *F* | *p* |
| --- | --- | --- | --- | --- | --- |
| Anchor | 10.27 | 1 | 10.27 | 4.57 | 0.033 |
| Debiasing | 11.56 | 2 | 5.78 | 2.57 | 0.078 |
| Interaction | 12.16 | 2 | 6.08 | 2.70 | 0.068 |
| Explained | 34.12 | 5 | 6.82 | 3.04 | 0.011 |
| Residual | 899.51 | 400 | 2.25 |  |  |
| Total | 15109 | 406 |  |  |  |

**Table 3. Effects of anchoring and debiasing techniques on evaluation of the service dimension.**

| Source | *SS* | *df* | *MS* | *F* | *p* |
| --- | --- | --- | --- | --- | --- |
| Anchor | 27.35 | 1 | 27.35 | 11.19 | 0.001 |
| Debiasing | 13.51 | 2 | 6.76 | 2.76 | 0.064 |
| Interaction | 3.56 | 2 | 1.78 | 0.73 | 0.48 |
| Explained | 44.70 | 5 | 8.94 | 3.66 | 0.003 |
| Residual | 977.88 | 400 | 2.45 |  |  |
| Total | 11414 | 406 |  |  |  |

**Table 4. Effects of anchoring and debiasing techniques on evaluation of the delivery dimension**

| Source | *SS* | *df* | *MS* | *F* | *p* |
| --- | --- | --- | --- | --- | --- |
| Anchor | 7.26 | 1 | 7.26 | 3.22 | 0.074 |
| Debiasing | 12.94 | 2 | 6.47 | 2.87 | 0.058 |
| Interaction | 4.82 | 2 | 2.41 | 1.07 | 0.35 |
| Explained | 25.16 | 5 | 5.03 | 2.23 | 0.050 |
| Residual | 902.06 | 400 | 2.26 |  |  |
| Total | 16309 | 406 |  |  |  |

**Table 5. Effects of anchoring and debiasing techniques on evaluation of the likelihood of choosing the same supplier.**

| Source | *SS* | *df* | *MS* | *F* | *p* |
| --- | --- | --- | --- | --- | --- |
| Anchor | 4404.85 | 1 | 4404.85 | 12.28 | 0.001 |
| Debiasing | 5228.55 | 2 | 2614.27 | 7.29 | 0.001 |
| Interaction | 2448.48 | 2 | 1224.24 | 3.41 | 0.034 |
| Explained | 12141.43 | 5 | 2428.29 | 6.77 | <0.0005 |
| Residual | 143445.09 | 400 | 358.61 |  |  |
| Total | 2113889 | 406 |  |  |  |

**Appendix 2 – Mann-Whitney Test Results (Study 1)**

| **Hypothesis Test Summary** | | | | |
| --- | --- | --- | --- | --- |
|  | Null Hypothesis | Test | Sig.^a,b^ | Decision |
| 1 | The distribution of evaluation on cost 1: lowest 9: highest is the same across categories of Experimental Condition. | Independent-Samples Mann-Whitney U Test | <.001 | Reject the null hypothesis. |
| 2 | The distribution of evaluation on quality 1: lowest 9: highest is the same across categories of Experimental Condition. | Independent-Samples Mann-Whitney U Test | .008 | Reject the null hypothesis. |
| 3 | The distribution of evaluation on service 1: lowest 9: highest is the same across categories of Experimental Condition. | Independent-Samples Mann-Whitney U Test | .036 | Reject the null hypothesis. |
| 4 | The distribution of evaluation on delivery 1: lowest 9: highest is the same across categories of Experimental Condition. | Independent-Samples Mann-Whitney U Test | .014 | Reject the null hypothesis. |
| 5 | The distribution of likelihood that you will have Supplier A as your supplier in future is the same across categories of Experimental Condition. | Independent-Samples Mann-Whitney U Test | <.001 | Reject the null hypothesis. |
| a. The significance level is .050. | | | | |
| b. Asymptotic significance is displayed. | | | | |

**Independent-Samples Mann-Whitney U Test**

**Evaluation on cost 1: lowest 9: highest across Experimental Condition**

| **Independent-Samples Mann-Whitney U Test Summary** | |
| --- | --- |
| Total N | 104 |
| Mann-Whitney U | 264.500 |
| Wilcoxon W | 1642.500 |
| Test Statistic | 264.500 |
| Standard Error | 151.591 |
| Standardized Test Statistic | -7.174 |
| Asymptotic Sig.(2-sided test) | <.001 |

| **Independent-Samples Mann-Whitney U Test Summary** | |
| --- | --- |
| Total N | 104 |
| Mann-Whitney U | 955.500 |
| Wilcoxon W | 2333.500 |
| Test Statistic | 955.500 |
| Standard Error | 148.430 |
| Standardized Test Statistic | -2.671 |
| Asymptotic Sig.(2-sided test) | .008 |

| **Independent-Samples Mann-Whitney U Test Summary** | |
| --- | --- |
| Total N | 104 |
| Mann-Whitney U | 1035.000 |
| Wilcoxon W | 2413.000 |
| Test Statistic | 1035.000 |
| Standard Error | 150.960 |
| Standardized Test Statistic | -2.100 |
| Asymptotic Sig.(2-sided test) | .036 |

| **Independent-Samples Mann-Whitney U Test Summary** | |
| --- | --- |
| Total N | 104 |
| Mann-Whitney U | 993.000 |
| Wilcoxon W | 2371.000 |
| Test Statistic | 993.000 |
| Standard Error | 145.996 |
| Standardized Test Statistic | -2.459 |
| Asymptotic Sig.(2-sided test) | .014 |

| **Independent-Samples Mann-Whitney U Test Summary** | |
| --- | --- |
| Total N | 104 |
| Mann-Whitney U | 718.500 |
| Wilcoxon W | 2096.500 |
| Test Statistic | 718.500 |
| Standard Error | 153.676 |
| Standardized Test Statistic | -4.122 |
| Asymptotic Sig.(2-sided test) | <.001 |
